# Short-Term Effectiveness of a Psychotherapeutic Intervention Model in Nursing for Ineffective Coping in GAD Outpatients: A Small-Sample Randomized Controlled Trial

**DOI:** 10.31083/AP51147

**Published:** 2026-10-14

**Authors:** Tuğba Aşkan, Birgül Özkan, Gülsüm Zuhal Kamış, Mustafa Uğurlu

**Affiliations:** ^1^Psychiatric Nursing, Educational Unit, Ankara Bilkent City Hospital, 06800 Ankara, Turkiye; ^2^Faculty of Health Sciences, Psychiatric Nursing, Ankara Yıldırım Beyazıt University, 06760 Ankara, Turkiye; ^3^Psychiatry Department, Ankara Bilkent City Hospital, 06800 Ankara, Turkiye

**Keywords:** coping skills, generalized anxiety disorder, nursing diagnosis, psychotherapeutic processes, self-esteem

## Abstract

**Background:**

Although nurse-delivered psychotherapeutic interventions have demonstrated benefits for individuals with anxiety disorders, no randomized controlled trial has evaluated the effectiveness of the Psychotherapeutic Intervention Model in Nursing for the nursing diagnosis of ineffective coping among psychiatric outpatients with generalized anxiety disorder (GAD). This study aimed to examine the short-term effectiveness of this model on the nursing-sensitive outcomes of coping and self-esteem.

**Methods:**

This single-blind randomized controlled trial was conducted among patients diagnosed with GAD. A total of 20 participants were randomized, of whom 17 completed the study. Seven of eight participants in the IG and eight of nine participants in the TAU group received pharmacotherapy, while participants in the IG additionally participated in a seven-week Psychotherapeutic Intervention Model in Nursing program. Data were collected at baseline (T0) and week 8 (T1) using information forms and the Nursing Outcomes Classification (NOC) Coping and Self-Esteem outcome measures. Statistical analyses included descriptive statistics, chi-square tests, independent-samples t tests, and repeated-measures general linear models. Statistical significance was set at *p* < 0.05.

**Results:**

Significant time × group interactions were observed for both NOC Coping and Self-Esteem outcomes. Participants in the IG demonstrated significantly greater improvements over time than those in the TAU group in coping (T0: 42.87 ± 6.03, T1: 76.75 ± 5.34, *p *< 0.001) and self-esteem (T0: 57.25 ± 7.29, T1: 78.62 ± 6.14, *p *< 0.001). In the TAU group, no significant changes were observed in coping (T0: 45.11 ± 6.70, T1: 45.33 ± 8.96, *p* > 0.05) or self-esteem (T0: 59.44 ± 8.63, T1: 58.89 ± 10.11, *p* > 0.05).

**Conclusions:**

This study provides preliminary evidence supporting the short-term effectiveness of the Psychotherapeutic Intervention Model in Nursing in improving NOC Coping and Self-Esteem outcomes among psychiatric outpatients with GAD and a nursing diagnosis of ineffective coping. Larger, multicenter randomized controlled trials with longer follow-up periods are warranted to confirm these findings.

**Clinical Trial Registration:**

This study is registered on ClinicalTrials.gov at https://clinicaltrials.gov/study/NCT06076746 (registration number: NCT06076746).

## Main Points

1. To our knowledge, this study is the first to evaluate the effectiveness of the Psychotherapeutic Intervention Model in Nursing for the nursing diagnosis of ineffective coping among outpatients diagnosed with Generalised Anxiety Disorder.
2. The findings provide preliminary evidence that this model can be implemented as an effective intervention to improve coping skills and self-esteem alongside treatment-as-usual.
3. The integration of nursing classifications in all stages of nursing care is important for grounding clinical practice in nursing knowledge.
4. The results of this study may contribute to the development of evidence-based psychiatric nursing practices and improve access to psychotherapeutic interventions.

## 1. Introduction

Generalized Anxiety Disorder (GAD) is one of the most prevalent mental disorders, with a lifetime prevalence of 3.7% [1]. It is characterized by persistent and excessive anxiety and worry that are difficult to control, often accompanied by physiological arousal and somatic tension, and affecting multiple domains of daily life, including health, finances, and routine activities [2,3]. In an effort to reduce distress and regain a sense of control, individuals with GAD frequently adopt rigid and repetitive behavioural strategies. However, when these strategies prove ineffective, anxiety may persist and negatively affect personal, occupational, and social functioning [4].

Individuals with GAD often believe that worrying facilitates effective coping with feared events or helps prevent them from occurring. When negative beliefs about worry develop, individuals may engage in reassurance-seeking and avoidance behaviours in an attempt to satisfy their perceived need to worry. These behaviours limit the use of alternative coping strategies and contribute to the maintenance of the anxiety cycle [5]. Consistent with this mechanism, individuals with GAD generally exhibit low levels of adaptive coping and tend to adopt passive or avoidant responses to stress, preferring to worry or ruminate rather than actively seeking solutions or support [6]. Moreover, evidence suggests that emotion-focused and avoidance-oriented coping styles are more prevalent than task-oriented coping among individuals with GAD. Although these strategies may provide temporary relief, they can reinforce maladaptive cognitive patterns, sustain anxiety, and reduce perceived self-efficacy. In contrast, task-oriented coping involves active problem-solving, cognitive restructuring, and engagement with stressors [7]. Therefore, strengthening coping skills represents an important therapeutic target in GAD, as it supports effective short-term stress management, promotes long-term emotional stability, and may help reduce the progression of anxiety disorders [8].

The combination of pharmacotherapy and psychological therapy for anxiety disorders has been shown to produce superior outcomes [9]; however, pharmacotherapy remains the preferred initial treatment due to cost and resource limitations [10]. In the absence of appropriate treatment, the condition may become chronic, leading to significant disability and financial burden [11]. While pharmacotherapy primarily reduces physiological arousal and anxiety symptoms [12], psychological interventions target the maladaptive cognitive and behavioural processes that sustain excessive worry and ineffective coping [13]. In this context, psychiatric nurses are well positioned to deliver psychotherapeutic interventions aimed at improving coping skills.

Evidence suggests that psychotherapeutic interventions delivered by psychiatric nurses are effective in the management of anxiety disorders. Amaral et al. (2022) [14] demonstrated that nurse-led therapeutic interventions improved anxiety self-control, reduced anxiety levels, and decreased the use of pharmacotherapy. These findings are consistent with previous evidence showing that psychiatric nurses possess the competencies in establishing therapeutic relationships and delivering psychotherapeutic interventions [15,16,17,18,19]. However, the literature describing standardized methodologies for implementing such interventions remains limited [20]. Therefore, structured models that standardize the content and sequence of psychotherapeutic nursing interventions are needed to support consistent and evidence-based clinical practice [18].

Within the nursing process, coping is systematically assessed and managed through the International Nursing Knowledge Association (NANDA-I) nursing diagnosis Ineffective Coping (00069), which provides the basis for planning individualized nursing interventions. NANDA-I defines maladaptive coping as “counterproductive cognitive and/or behavioral efforts to manage a stressful or unpleasant situation” [21]. Enhancing individuals’ coping skills is essential for the effectiveness of nursing interventions [22]. Accordingly, the nursing diagnosis of Ineffective Coping provides the foundation for establishing standardized linkages among nursing diagnoses, interventions, and outcomes within the nursing process.

Standard classification systems developed by professional nursing organizations are used to support scientific and evidence-based nursing care within the nursing process [4]. NANDA-I [21], the Nursing Interventions Classification (NIC) [23], and the Nursing Outcomes Classification (NOC) [24] are among the most widely used standardized nursing language systems [25]. These systems are organized within a hierarchical taxonomic framework in which each nursing diagnosis, intervention, and outcome is assigned a unique standardized code to facilitate consistent documentation, communication, and clinical decision-making. Standardized nursing language linkages integrate nursing diagnoses, interventions, and outcomes into a structured framework for planning, implementing, and evaluating nursing care. Nevertheless, further clinical research is needed to establish the effectiveness of these standardized linkages and strengthen the evidence supporting their use in clinical practice [26].

In response to this need, Sampaio et al. (2017) [18] developed the Psychotherapeutic Intervention Model in Nursing, based on nursing knowledge, to guide and systematize nursing psychotherapeutic interventions through standardized linkages among nursing diagnoses, interventions, and outcomes. The model structure is based on NANDA-I/International Classification for Nursing Practice (ICNP), NIC, and NOC. The interventions adopt an integrated approach, combining techniques derived from different schools of psychotherapy. The model is designed to provide guidance throughout all stages of the nursing process and to offer a framework applicable across diverse clinical settings [18]. In evaluating the effectiveness of the Psychotherapeutic Intervention Model in Nursing for the “anxiety” nursing diagnosis in individuals with GAD, Sampaio et al. (2018) [27] found that the model demonstrated short-term effectiveness in reducing anxiety levels and improving anxiety self-control.

Despite the central role of ineffective coping in GAD, no randomized controlled trial has evaluated the Psychotherapeutic Intervention Model in Nursing for the NANDA-I nursing diagnosis of Ineffective Coping among psychiatric outpatients with GAD. Therefore, the present study aimed to fill this gap by evaluating the effectiveness of this model in improving the NOC outcomes of Coping and Self-Esteem in this population. The study was conducted assuming that participants in the intervention group would demonstrate greater improvements in NOC Coping and NOC Self-Esteem from baseline to post-intervention compared to the participants receiving treatment-as-usual (TAU), reflecting a significant time x group interaction.

## 2. Materials and Methods

### 2.1 Study Design and Participants

 This study was conducted as a single-blind randomized controlled trial in the psychiatric outpatient clinic of Ankara Bilkent City Hospital in Turkey. The study population consisted of patients diagnosed with GAD who were receiving outpatient follow-up care at the clinic. As no previous studies had reported data on the primary outcome (coping) variable the effect size reported by Sampaio et al. (2018) [27] was used to calculate the sample size.

The sample size was calculated using G*Power version 3.1.9.6 (Heinrich-Heine-Universität Düsseldorf, Düsseldorf, Germany) based on a mixed-design repeated-measures ANOVA with a 2 × 2 design. The type I error probability (α) was set at 0.05, and statistical power (1 – β) was set at 0.95. In the study by Sampaio et al. (2018) [27], Cohen’s d was reported as 1.11, corresponding to an f value of 0.55 after conversion from η^2^. Based on this calculation, the minimum required sample size was 14 participants (7 per group). Considering potential dropouts due to the seven-week intervention period, a total of 20 participants were planned for enrollment, with 10 participants assigned to each group.

During participant recruitment, every third patient diagnosed with GAD in the outpatient clinic was referred to the researcher by the physician. Eligible participants were randomly assigned to either the intervention or TAU group using simple randomization in SPSS 28 (IBM Corp., Armonk, NY, USA). To ensure reproducibility of the randomization procedure, a fixed random seed was used. The randomization sequence was generated and maintained by a psychiatrist with expertise in statistics who was not involved in participant recruitment, outcome assessment, or intervention delivery. Group assignments were concealed from the researcher, and allocation information was obtained from the psychiatrist only after each participant had been enrolled. A Consolidated Standards of Reporting Trials (CONSORT) diagram was used to illustrate participant flow throughout the study [28] (Fig. 1).

**Fig. 1.**
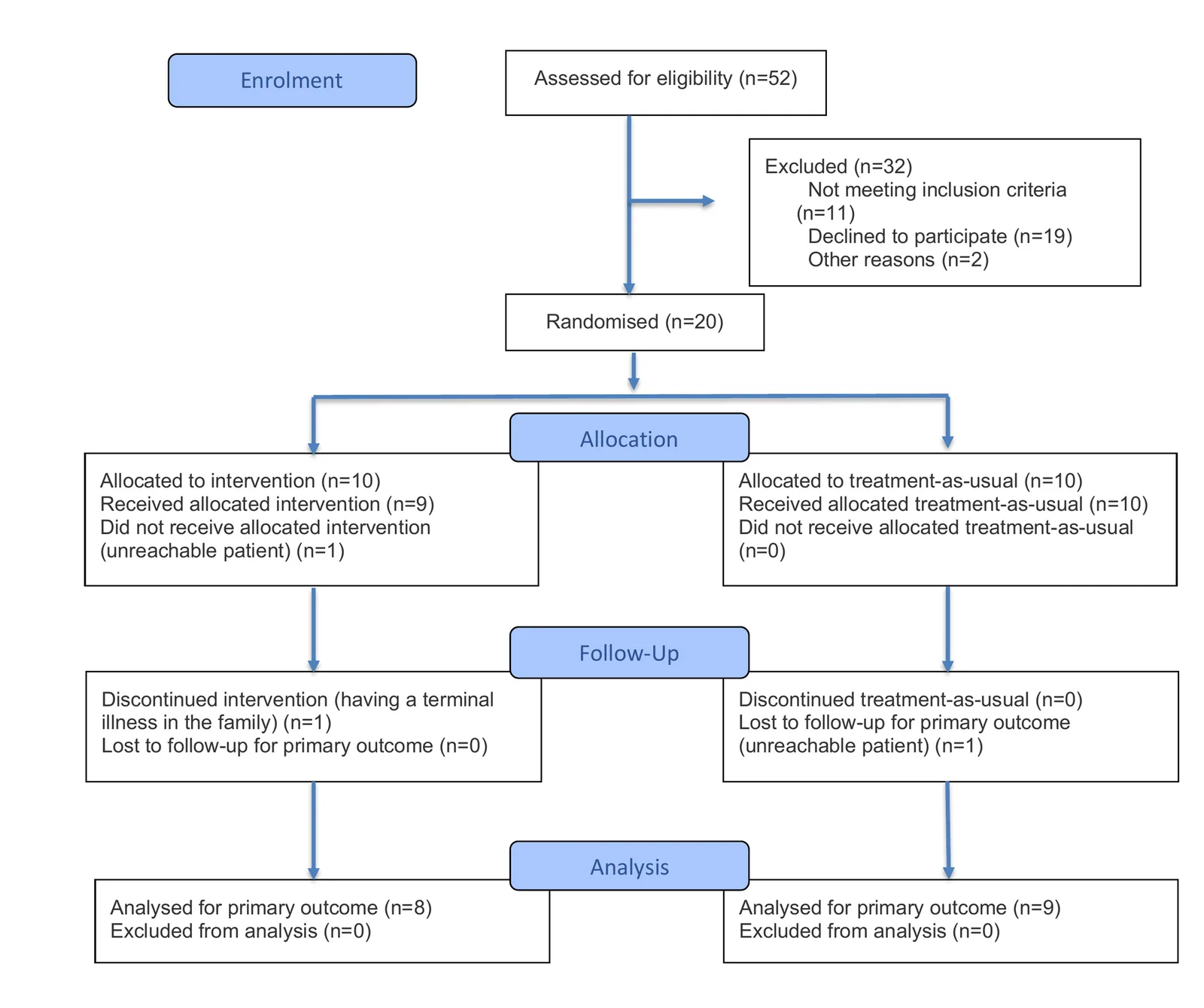
**CONSORT 2025 flow diagram**. CONSORT, Consolidated Standards of Reporting Trials.

Psychiatric assessments and medical treatment provided by psychiatrists were continued as usual for participants in both the TAU and the intervention groups. Follow-up visits and psychiatric evaluations, including assessments of medication effectiveness and potential side effects, were performed during routine outpatient clinic visits and were not collected as study data. Participants in both groups were assessed before and after the intervention period using Information Form-1, Information Form-2, and the NOC outcomes of Coping (1302) and Self-Esteem (1205). In addition to routine treatment, participants in the intervention group received the Psychotherapeutic Intervention Model in Nursing.

Inclusion criteria were as follows: age between 18–64 years, literacy (defined as having acquired basic reading and writing skills, regardless of completion of primary education), currently receiving follow-up care at the psychiatric outpatient clinic, diagnosis of GAD, and the presence of at least one defining characteristic of ineffective coping according to the nursing process.

Defining characteristics of ineffective coping included altered affective responsiveness, altered attention, an altered communication pattern, difficulty organizing information, fatigue, frequent illness, impaired ability to ask for help, impaired ability to attend to information, impaired ability to manage situations, impaired ability to meet basic needs or fulfill role expectations, inadequate follow-through with goal-directed behavior, inadequate problem-solving skills, altered sleep-wake cycle, inadequate sense of control, risk-taking behavior (substance misuse and destructive behaviors toward self or others were excluded).

Exclusion criteria were as follows: dementia, psychotic spectrum disorders, cognitive impairment, an altered level of consciousness, psychomotor agitation, moderate or severe intellectual disability, suicidal ideation, suicide attempt within the past month, current substance misuse or destructive behaviors toward self or others, and ongoing psychological counseling with a clinical psychologist or psychiatrist.

### 2.2 Intervention

The establishment of NANDA-I, NOC, and NIC linkages (NNN) for Ineffective Coping (00069) was informed by clinical reasoning and the framework proposed by Johnson et al. (2011) [26]. Psychotherapeutic interventions corresponding to the NOC outcomes Coping and Self-Esteem included Counseling (5240), Active Listening (4920), Relaxation Therapy (6040), Anxiety Reduction (5820), Cognitive Restructuring (4700), Coping Enhancement (5230), Decision-Making Support (5250), Sleep Enhancement (1850), Crisis Intervention (6160), Impulse Control Training (4370), Behavior Modification (4360), Self-Esteem Enhancement (5400), Emotional Support (5270), and Assertiveness Training (4340) [23].

The researcher selected interventions based on individual patient assessments. Consistent with the model’s integrated approach, which combines psychodynamic, cognitive-behavioral, and humanistic techniques, NIC interventions were implemented accordingly. The intervention program consisted of seven weekly individual sessions, with one session delivered per week, each lasting 45–60 minutes. The model structure is presented in Fig. 2 (Ref. [18]). Following completion of the baseline assessments, the Psychotherapeutic Intervention Model in Nursing was implemented between 14 October and 27 December 2024.

**Fig. 2.**
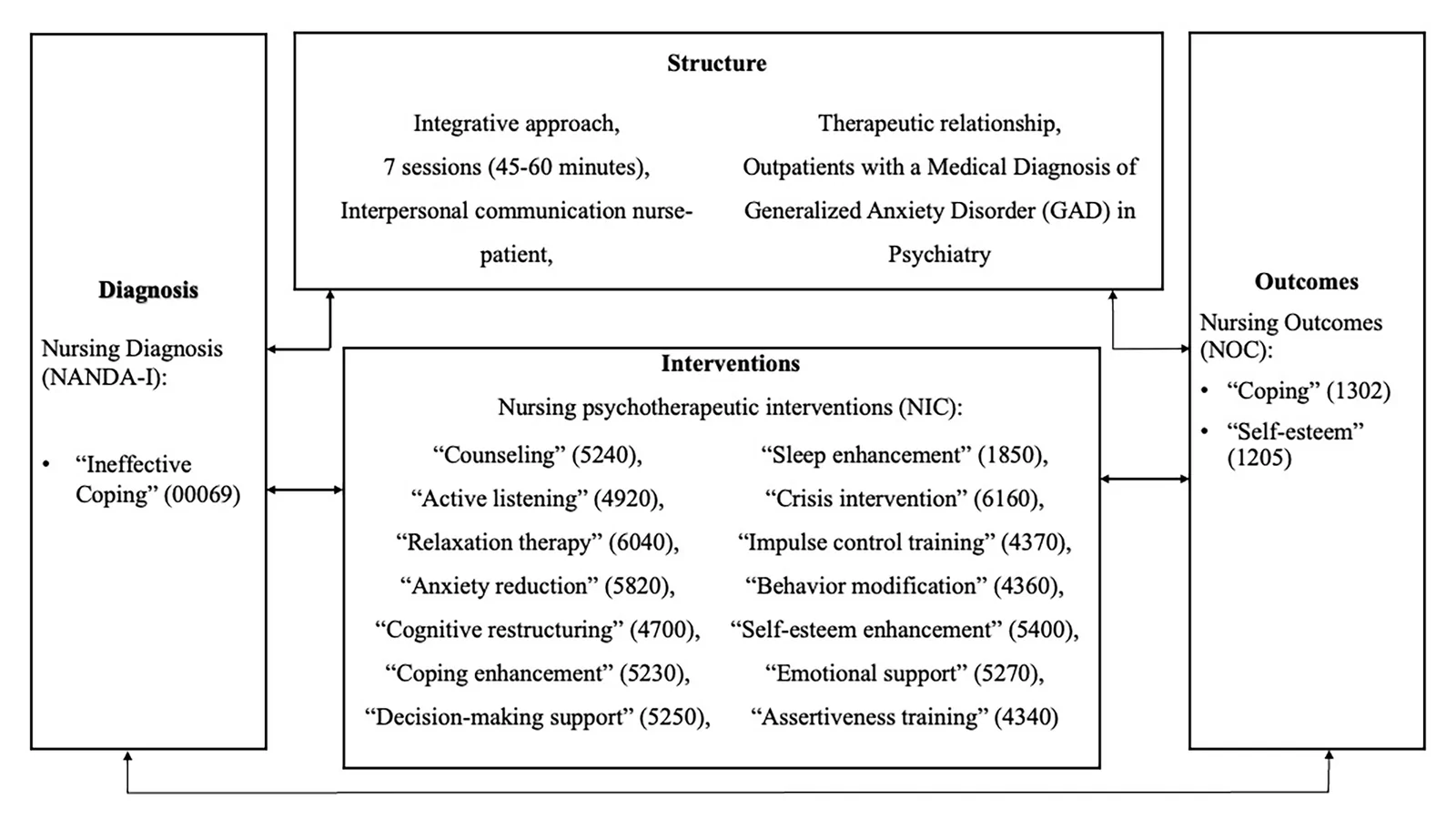
**Psychotherapeutic intervention model in nursing for the diagnosis of ineffective coping (based on the Sampaio et al. (2017) [18])**. NANDA-I, International Nursing Knowledge Association; NOC, Nursing Outcomes Classification; GAD, Generalized Anxiety Disorder; NIC, Nursing Interventions Classification.

The main objectives of the sessions were to:

- explore the causes of anxiety,
- provide education on common anxiety symptoms,
- train participants in effective coping strategies,
- discuss activities to reduce anxiety,
- evaluate perceived progress and strategy effectiveness.

The intervention was delivered individually by the first author, a psychiatric nurse and PhD candidate who had received formal psychotherapy training and had prior experience in delivering psychotherapeutic interventions. The intervention was implemented in accordance with the Psychotherapeutic Intervention Model in Nursing for the Diagnosis of Ineffective Coping protocol developed for this study (Fig. 2). Participant attendance was monitored throughout the intervention period, and attendance records were maintained for each session. Of the nine participants who initiated the intervention, eight (88.9%) completed all seven intervention sessions, while one participant (11.1%) completed six of the seven sessions. The overall attendance rate was 98.4%. Furthermore, regular supervision was provided throughout the intervention by an academic in psychiatric nursing and a psychiatrist, both of whom were members of the research team.

### 2.3 Data Collection

Eligible patients were referred to the researcher by physicians, after which inclusion and exclusion criteria were assessed. Participation was voluntary, and both written and verbal informed consent were obtained from all participants. Baseline assessments were conducted by an independent evaluator who was blinded to group allocation. The psychiatric nurse responsible for outcome assessment received two training sessions on the administration and scoring of the NOC outcomes.

Baseline data collection included Information Form-1 (17 items assessing demographic characteristics and psychiatric history), Information Form-2 (perception of coping), and Turkish versions of NOC outcomes Coping (1302) and Self-Esteem (1205). Post-intervention data collection included NOC outcomes and Information Form-2. Each assessment took approximately 60 minutes.

### 2.4 Measurement Instruments

#### NOC Outcomes Coping (1302) and Self-Esteem (1205)

The NOC was developed in 1991 by a research team at the University of Iowa with the aim of establishing a standardized classification system for evaluating patient status before and after nursing interventions. The sixth edition (2018) includes a total of 540 outcomes organized within a taxonomic structure comprising seven domains and 31 classes, in which each outcome is assigned a standardized code [24].

NOC outcomes are measured using 5-point Likert-type scales, where 1 indicates the least desirable condition and 5 represents the most desirable condition. Outcome evaluation is performed by calculating mean scores based on relevant indicators, which may also be used individually when necessary [24].

In the present study, the NOC outcomes Coping (1302) and Self-Esteem (1205) were used. The numbers 1302 and 1205 represent the standardized NOC codes assigned to these outcomes within the NOC taxonomy. Coping is defined as “personal actions to manage stressors that tax an individual’s resources” and includes 19 indicators. Self-Esteem, defined as “personal judgment of self-worth”, consists of 20 indicators [24].

The Turkish validity and reliability study of the NOC outcomes Coping and Self-Esteem was conducted in a sample of psychiatric outpatients. The Turkish version of the Coping outcome includes 19 indicators and a single dimension, with total scores ranging from 19 to 95. Inter-rater reliability demonstrated ICC values of 0.985 for single measurements and 0.993 for average measurements. Internal consistency was acceptable, with Cronbach’s alpha of 0.815 and McDonald’s omega of 0.830 [29].

The Turkish version of the Self-Esteem outcome consists of 19 indicators and three subdimensions: Self-Confidence, Social Approval, and Internal Value/Negative Self-Evaluation. Total scores range from 19 to 95, while subscale scores range from 9–45, 5–25, and 5–25, respectively. Inter-rater reliability showed intraclass correlation coefficient (ICC) values of 0.984 (single measurements) and 0.992 (average measurements). Internal consistency was also satisfactory, with a Cronbach’s alpha coefficient of 0.832 and a McDonald’s omega coefficient of 0.841 [29].

### 2.5 Statistical Analyses

Data were analyzed using IBM SPSS Statistics 28.0. Descriptive statistics were used to summarize participant characteristics. Chi-Square tests and independent-samples *t*-tests were performed to compare baseline group characteristics. Primary analyses were conducted using a per-protocol approach, including only participants who completed both baseline and post-intervention assessments. An intention-to-treat analysis was not performed because complete outcome data were not available for all participants who withdrew after randomization. Repeated-measures general linear models (GLM), separately for the NOC Coping and NOC Self-Esteem outcomes, were used to examine the main effects of time (baseline vs. post-intervention) and group (intervention vs. TAU), as well as the time × group interaction. Between-group standardized effect sizes were estimated using Hedges’ *g* for both baseline and post-intervention comparisons. Statistical significance was defined as *p* < 0.05.

## 3. Results

### 3.1 Characteristics of the Sample

Table 1 presents the descriptive characteristics of the participants. Of the 20 randomized participants, 8 were in the intervention and 9 were included in the TAU group in the final analysis. The mean age was 38.50 ± 10.60 years in the intervention group and 40.00 ± 9.62 years in the TAU group. The majority of participants were female (87.5% in the intervention group and 88.8% in the TAU group), and the majority were married (75.0% and 55.5%, respectively). A physical illness was reported by 37.5% of participants in the intervention group and 33.3% of those in the TAU group. All participants met the diagnostic criteria for generalized anxiety disorder, and most were receiving pharmacotherapy (87.5% in the intervention group and 88.8% in the TAU group), predominantly antidepressants. Statistical comparisons revealed no significant differences between the intervention and TAU groups across baseline characteristics (*p* > 0.05).

**Table 1.** **Comparison of mean scores for participants’ descriptive characteristics between groups (*N* = 17)**.

Descriptive characteristics		IG (*n* = 8) *n* (%)	TAU (*n* = 9) *n* (%)	*p*
Gender*	Female	7 (87.50)	8 (88.88)	0.929
	Male	1 (12.50)	1 (11.11)	
Marital status	Married	6 (75.00)	5 (55.55)	0.704
	Single	1 (12.50)	2 (22.22)	
	Other	1 (12.50)	2 (22.22)	
Education level*	Primary school	0 (0.00)	2 (22.22)	0.096
	Middle school	1 (12.50)	2 (22.22)	
	High school	1 (12.50)	3 (33.33)	
	University	6 (75.00)	1 (11.11)	
	Postgraduate education	0 (0.00)	1 (11.11)	
Perceived socioeconomic status*	Low	2 (25.00)	3 (33.33)	0.706
	Moderate	6 (75.00)	6 (66.66)	
	High	0 (0.00)	0 (0.00)	
Presence of physical illness*	Yes	3 (37.50)	3 (33.33)	0.627
	No	5 (62.50)	6 (66.67)	
Physical illness*	Asthma	1 (12.50)	1 (11.11)	
	Heart disease	1 (12.50)	0 (0.00)	
	Hypertension and chronic viral hepatitis	1 (12.50)	0 (0.00)	
	Diabetes	0 (0.00)	1 (11.11)	
	Epilepsy	0 (0.00)	1 (11.11)	
Psychiatric diagnosis*	Generalized anxiety disorder	8 (100.00)	9 (100.00)	
	Depressive episode***	1 (12.50)	2 (22.22)	
	Adjustment disorder***	1 (12.50)	0 (0.00)	
	Obsessive–compulsive disorder***	0 (0.00)	1 (11.1)	
Use of pharmacotherapy*	Yes	7 (87.50)	8 (88.88)	0.929
	No	1 (12.50)	1 (11.11)	
Pharmacotherapy type*	Antidepressant	7 (87.50)	8 (88.88)	
	Benzodiazepine	1 (12.50)	1 (11.1)	
	Antipsychotic		1 (11.1)	
		*M* ± *SD* (Minimum–Maximum)		
Age**		38.50 ± 10.60 (23–54)	40.00 ± 9.62 (27–55)	0.763
Baseline perceived coping**		4.37 ± 1.77 (1–6)	5.55 ± 2.74 (1–9)	0.315
Post-intervention perceived coping**		7.75 ± 1.91 (5–10)	6.44 ± 2.70 (2–10)	0.273

*chi-square test; **Independent-samples *t* test; ***comorbid diagnoses in addition to GAD; *M* ± *SD* = mean ± standard deviation. IG, intervention group; TAU, treatment-as-usual.

Three participants withdrew after randomization. Two participants in the intervention group discontinued participation: one before baseline assessment because the allocated intervention could not be initiated, and the other during week 6 because of a family member developed a terminal illness. One participant in the TAU group was lost to follow-up because contact could not be established. Participants who withdrew had a mean age of 43.00 ± 1.73 years, and all were female. Participants who completed the study had a mean age of 39.29 ± 9.78 years, of whom 88.2% were female. Complete baseline clinical outcome data were not available for all participants who withdrew after randomization. Based on the limited available data, no statistically significant differences were observed in basic demographic characteristics between study completers and participants who withdrew.

The mean baseline coping perception score in the intervention group was 4.37 ± 1.77 (range: 1–6), which increased to 7.75 ± 1.91 (range: 5–10) at post-intervention. In the TAU group, the mean baseline score was 5.55 ± 2.74 (range: 1–9), increasing to 6.44 ± 2.70 (range: 2–10) at post-intervention (Table 1).

Regarding psychiatric treatment history, none of the participants had a history of inpatient psychiatric treatment. Previous psychiatric consultations initiated through medical departments were reported by 12.5% of participants in the intervention group and 33.3% of those in the TAU group. A history of psychotherapy or counseling was reported by 37.5% of participants in the intervention group and 22.2% of those in the TAU group. Most participants (87.5% in the intervention group and 88.8% in the TAU group) had previously considered receiving psychotherapy or counseling, and all participants reported believing in its benefits (Table 2).

**Table 2.** **Between-group comparison of participants’ psychiatric treatment characteristics (*N* = 17)**.

Psychiatric treatment characteristics		IG (*n* = 8) *n* (%)	TAU (*n* = 9) *n* (%)
Hospitalization for psychiatric treatment	Yes	0 (0.00)	0 (0.00)
	No	8 (100.00)	9 (100.00)
Receiving psychiatric consultation from a medical department	Yes	1 (12.50)	3 (33.33)
	No	7 (87.50)	6 (66.66)
Previous experience with psychotherapy or counseling	Yes	3 (37.50)	2 (22.22)
	No	5 (62.50)	7 (77.77)
Considering psychotherapy or counseling	Yes	7 (87.50)	8 (88.88)
	No	1 (12.50)	1 (11.11)
Belief in the effectiveness of psychotherapy or counseling	Yes	8 (100.00)	9 (100.00)
	No	0 (0.00)	0 (0.00)

### 3.2 Effect of the Intervention

Table 3 presents the descriptive statistics and repeated-measures GLM results for the NOC outcomes Coping and Self-Esteem. For NOC Coping, the analysis revealed a significant main effect of time, *F*(1, 15) = 163.98,* p* < 0.001, partial *η*^2^ = 0.916, a significant main effect of group, *F*(1, 15) = 21.99, *p* < 0.001, partial *η*^2^ = 0.594, and a significant time × group interaction, *F*(1, 15) = 159.73, *p *< 0.001, partial *η*^2^ = 0.914. Coping scores increased substantially in the intervention group from baseline to post-intervention, whereas scores in the TAU group remained relatively unchanged. The between-group effect size was small at baseline (Hedges’ *g* = –0.33) and became very large at post-intervention, favoring the intervention group (Hedges’ *g* = 3.98). Fig. 3A illustrates changes in NOC Coping scores over time.

**Table 3.** **Descriptive statistics and repeated-measures GLM results for NOC outcomes**.

		T0 *M* ± *SD*	T1 *M* ± *SD*	Hedges’ *g* at T0	Hedges’ *g* at T1	Time effect, *F*(1, 15), *p*, *η*p^2^	Group effect, *F*(1, 15), *p*, *η*p^2^	Time × Group effect, *F*(1, 15), *p*, *η*p^2^
NOC coping	IG	42.87 ± 6.03	76.75 ± 5.34	–0.33	3.98	163.98, <0.001, 0.916	21.99, <0.001, 0.594	159.73, <0.001, 0.914
	TAU	45.11 ± 6.70	45.33 ± 8.96					
NOC self-esteem	IG	57.25 ± 7.29	78.62 ± 6.14	–0.26	2.21	199.40, <0.001, 0.930	4.94, 0.042, 0.248	221.25, <0.001, 0.937
	TAU	59.44 ± 8.63	58.89 ± 10.11					

T0, baseline; T1, post-intervention; *M*, mean; *SD*, standard deviation; *η*p^2^, partial eta squared. Hedges’ *g* values represent between-group standardized mean differences and were calculated as IG minus TAU. Negative baseline values indicate slightly lower baseline scores in the intervention group. The time × group effect represents the primary test of differential change over time between groups.

**Fig. 3.**
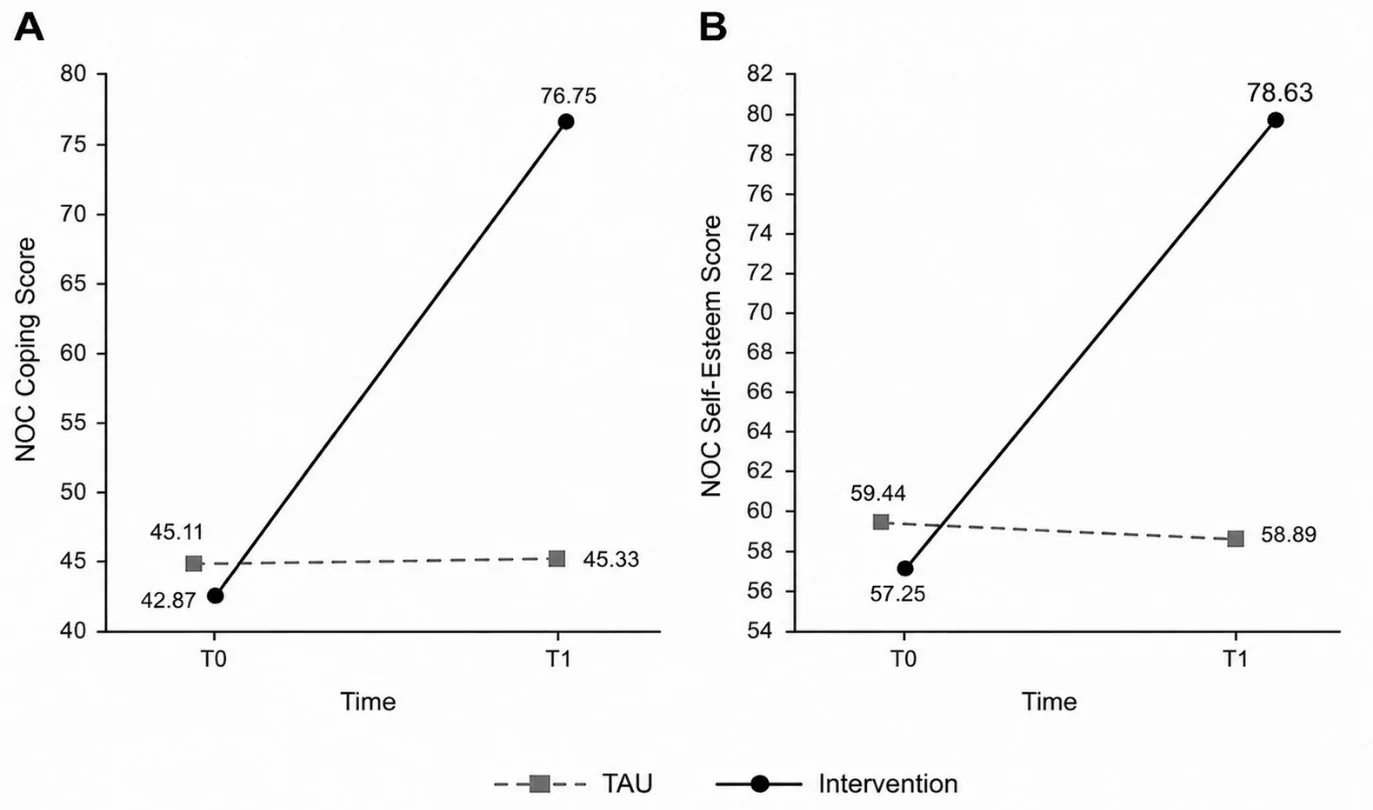
**Changes in NOC outcome scores from baseline (T0) to post-intervention (T1) by group**. (A) NOC Coping score. (B) NOC Self-Esteem score.

For NOC Self-Esteem, the analysis also revealed a significant main effect of time, *F*(1, 15) = 199.40, *p* < 0.001, partial *η*^2^ = 0.930, a significant main effect of group, *F*(1, 15) = 4.94, *p* = 0.042, partial *η*^2^ = 0.248, and a significant time × group interaction, *F*(1, 15) = 221.25, *p* < 0.001, partial *η*^2^ = 0.937. Self-esteem scores increased markedly in the intervention group, while scores in the TAU group showed no significant improvement. The between-group effect size was small at baseline (Hedges’ *g* = –0.26) and large at post-intervention, favoring the intervention group (Hedges’* g* = 2.21). Fig. 3B illustrates changes in NOC Self-Esteem scores over time.

Intra-rater reliability analyses demonstrated good agreement for the NOC Coping outcome and excellent agreement for the NOC Self-Esteem outcome. For the NOC Coping outcome, the ICC was 0.749 (95% CI [0.194, 0.938], *F*(8, 8) = 6.318, *p* = 0.009). For the NOC Self-Esteem outcome, the ICC was 0.968 (95% CI [0.872, 0.993], *F*(8, 8) = 57.608, *p* < 0.001). In addition, before the study, the blinded outcome assessor had received training in the use of NOC outcomes as part of a previous research project. In the Turkish adaptation study of the NOC outcomes, operational definitions were developed for each outcome indicator and its corresponding Likert-scale rating to standardize the scoring procedure [29]. The same standardized rating framework was used in the present study.

## 4. Discussion

The present study demonstrates the short-term positive effects of the Psychotherapeutic Intervention Model in Nursing for the nursing diagnosis of ineffective coping among outpatient psychiatric patients diagnosed with GAD. The findings suggest that implementing this model alongside pharmacotherapy represents an effective approach for improving coping and self-esteem. In contrast, usual treatment and care alone, including pharmacotherapy when applicable, did not produce meaningful improvements in these domains. As no prior studies have evaluated the effects of this model on coping and self-esteem in this population, interpretation of the findings is limited by the lack of comparable evidence. Furthermore, due to the small sample size, these findings should be regarded as preliminary and interpreted cautiously until confirmed in larger randomized studies.

Statistical comparisons between the intervention and TAU groups revealed no significant baseline differences, indicating overall group homogeneity. However, although the difference in baseline coping perception scores between the groups did not reach statistical significance, participants in the intervention group had numerically lower scores than those in the TAU group. This baseline numerical difference should be taken into consideration when interpreting the findings, as it may have influenced the changes observed over time. Nevertheless, the repeated-measures GLM evaluated the effects of time and group, as well as the time × group interaction, to assess differential changes between the groups over time.

However, clinician-rated NOC Coping scores demonstrated significant improvements, highlighting a discrepancy between self-reported and clinician-assessed outcomes. This finding aligns with evidence from a meta-analysis indicating that self-report measures in GAD often yield smaller effect sizes compared to clinician-rated instruments [30]. Similarly, a study conducted in Spain examining perceptions of individuals with GAD reported that some participants attributed the course of the disorder to external factors, associating flare-ups with stressful events, while others attributed recovery to their coping behaviours and healthcare support [31]. Coping strategies identified in that study included seeking external support and reassurance-seeking behaviours, which may have influenced self-reported coping perception scores in the study.

The findings supported the coping-related hypotheses, demonstrating a significant time × group interaction, a significant improvement in coping scores within the intervention group, and higher post-test coping scores compared with the TAU group. The observed improvement in coping may be explained by the fact that the intervention directly targeted the defining characteristics of the NANDA-I diagnosis of Ineffective Coping. These results suggest that NIC interventions such as Coping Enhancement, Relaxation Therapy, Behavior Modification, Cognitive Restructuring, Anxiety Reduction, Crisis Intervention, and Assertiveness Training can effectively develop coping skills. These interventions may have improved coping by increasing participants’ awareness of maladaptive coping responses, facilitating more adaptive cognitive appraisal of stressful situations and perceived threats, reducing anxiety-related physiological arousal, and promoting the acquisition and use of adaptive problem-focused and emotion-focused coping strategies [8,13,32]. This finding aligns with studies showing that combining cognitive behavioral therapy (CBT) with pharmacotherapy improves problem-focused coping, distraction, and social support scores, whereas pharmacotherapy alone does not significantly enhance effective coping [33]. Similarly, previous evidence has shown that problem-solving and cognitive reappraisal are associated with more adaptive coping, whereas avoidance-oriented strategies are linked to greater psychopathology among individuals with anxiety disorders [34]. The present findings are also consistent with studies reporting that nurse-administered psychoeducation programs improve coping, psychological well-being, and anxiety levels in patients with GAD [35]. Likewise, in the study by Sampaio et al. (2018) [27], combining the Psychotherapeutic Intervention Model in Nursing with pharmacotherapy resulted in improvements in anxiety self-control, whereas pharmacotherapy improved only psychomotor anxiety without affecting problem-solving or anxiety self-control scores. These findings underscore the clinical significance of psychotherapeutic interventions in enhancing coping mechanisms.

A meta-analysis examining psychotherapy for GAD reported that psychotherapeutic interventions have moderate-to-large effect sizes, whereas pharmacotherapy exhibits small effect sizes, suggesting that symptom alleviation alone is insufficient for developing new coping strategies [36]. Individuals with anxiety disorders often experience excessive anxiety and avoid perceived internal or external threats [37], which may limit opportunities to test maladaptive beliefs and develop adaptive coping strategies. Pharmacotherapy may indirectly facilitate coping by reducing anxiety symptoms and physiological arousal, thereby enabling individuals to engage more effectively with stressful situations [12,38,39]. However, pharmacotherapy alone does not explicitly teach adaptive coping strategies, modify maladaptive cognitive appraisals, or provide opportunities to practice new behavioral responses. Consistent with this distinction, although a meta-analysis reported that pharmacological treatments demonstrated larger overall effect sizes than psychotherapeutic interventions in reducing GAD symptom severity [1], the primary outcomes of the present study were nursing-related functional outcomes rather than symptom reduction. Therefore, the observed improvement in NOC Coping may reflect the intervention’s specific focus on enhancing coping through targeted NIC interventions rather than reducing symptoms alone.

The findings also supported the self-esteem-related hypotheses, demonstrating a significant time × group interaction, a significant increase in self-esteem scores within the intervention group, and higher post-test self-esteem scores compared with the TAU group. The intervention incorporated NIC interventions such as Cognitive Restructuring, Self-Esteem Enhancement, and Assertiveness Training. Improvements in self-esteem may be attributable to these intervention components, which were specifically designed to strengthen positive self-perceptions by helping participants identify and modify maladaptive self-beliefs, develop more adaptive self-evaluations, and express themselves more confidently in interpersonal situations. In addition, NIC interventions such as Counseling, Active Listening, and Emotional Support provided participants with opportunities to express emotions, receive constructive feedback, and experience successful coping within a supportive therapeutic environment. Improvements in self-esteem may also be explained by participants’ repeated experiences of successfully applying newly acquired coping strategies. Such experiences may strengthen perceptions of competence and personal control, both of which are recognized contributors to self-esteem [40].

The present findings also align with studies showing that psychoeducation and relaxation-based interventions increase perceived self-efficacy among patients with anxiety disorders [41]. This emphasizes the value of increasing patients’ awareness and skills for managing anxiety effectively.

Traditional biomedical or psychiatric treatments may inadequately address self-esteem, highlighting the need for psychotherapeutic interventions. Cognitive-behavioral techniques targeting dysfunctional thought patterns may improve self-esteem by modifying maladaptive cognitions [42]. These findings are consistent with previous studies demonstrating the effectiveness of nurse-led CBT programs and other psychotherapeutic interventions targeting self-esteem improvement [43,44,45,46].

It is also possible that some of the observed improvements were attributable, at least in part, to non-specific intervention effects. Increased therapeutic attention and contact time throughout the seven-session intervention, participants’ positive expectations regarding the intervention, and the therapeutic alliance established between the psychiatric nurse and participants may have contributed to the observed improvements beyond the effects of the specific NIC interventions delivered during the intervention.

Regarding feasibility, the dropout rate was 20% in the intervention group and 10% in the TAU group, suggesting that the intervention was both acceptable and feasible. Previous studies evaluating the Psychotherapeutic Intervention Model in Nursing reported a dropout rate of 31%, while nurse-led CBT studies reported dropout rates of approximately 25% [27,47]. Setting a predefined session duration and informing participants in advance may have contributed to improved adherence [48]. Additionally, participants’ belief in the benefits of psychotherapy may have supported sustained motivation throughout the intervention period.

### Strengths and Limitations

A key strength of this study is the use of clinician-rated NOC outcomes rather than self-reported measures. Outcome assessments were performed by a psychiatric nurse blinded to group assignments; randomisation was overseen by a psychiatrist with expertise in statistics, and patient eligibility was assessed by a psychiatric nurse. These procedures are notable strengths that mitigate detection, selection and allocation bias.

Due to the nature of the intervention, participants and the intervention provider could not be blinded. Awareness of group assignment may have influenced participants’ attitudes, motivation, engagement, and reported behaviors, potentially introducing performance and expectancy biases. NOC outcomes were assessed through structured interviews and clinical observations by a blinded assessor. Nevertheless, improvements should be interpreted with consideration of the potential influence of non-specific intervention effects. Moreover, all assessments were conducted by a single assessor; thus, intra-rater reliability was evaluated, precluding inter-rater reliability assessment and potentially limiting measurement consistency.

Formal intervention fidelity procedures, such as independent session reviews, fidelity checklists, or audio/video-recorded evaluations, were not implemented. Although the intervention followed a structured protocol with regular supervision, this may limit conclusions regarding intervention consistency and reproducibility.

Reliance on clinical decisions to select psychotherapeutic interventions based on NNN linkages represents another limitation. Furthermore, the control condition of usual treatment and care did not allow continuous monitoring of pharmacotherapy adherence, limiting direct comparisons.

Based on the calculated minimum sample size of 14 participants (7 per group) and an anticipated attrition rate of 40%, the study should have enrolled 24 participants (12 per group). However, only 20 participants were randomized, and the study was ultimately completed with 17 participants. This smaller-than-necessary final sample size is a limitation of the study and may have reduced the statistical power and precision of the findings. As no comparable randomized controlled trial using coping as the primary outcome was identified during study planning, the effect size for sample size estimation was derived from a psychotherapeutic intervention study assessing anxiety-related outcomes.

The large time × group effects and post-test between-group standardized differences indicated substantial statistical effects. Although these findings suggest potential clinical value, the lack of an established minimal clinically important difference (MCID) for the NOC outcomes means that the clinical interpretation of these findings should be considered preliminary and confirmed in larger, adequately powered randomized controlled trials.

The analyses were conducted using a per-protocol approach because outcome data were unavailable for the three participants who withdrew after randomization. Although their reasons for withdrawal appeared unrelated to treatment response, their exclusion may have introduced attrition bias; if these participants had experienced less improvement, the intervention effects may have been overestimated. Future studies should implement intention-to-treat analyses using multiple imputation or other robust methods for handling missing outcome data whenever feasible. The absence of clinical data for individuals who declined participation may also have limited assessment of the representativeness of the study sample.

Finally, the single-center convenience sample limits generalizability of the findings, and the absence of long-term follow-up precludes conclusions regarding sustained effects. Therefore, these findings should be interpreted with caution until replicated in larger, adequately powered, multicenter studies with more heterogeneous samples.

During the study, NANDA-I and NOC taxonomies were updated: “Ineffective coping” was revised to “maladaptive coping”, and some NOC outcomes were modified [21,49,50]. Therefore, Future applications and comparisons should consider these definitional and classification changes when interpreting the comparability of findings.

## 5. Conclusions

The present study provides preliminary evidence for the short-term effectiveness of the Psychotherapeutic Intervention Model in Nursing in enhancing coping and self-esteem among outpatients with GAD diagnosed with ineffective coping. It contributes to the development and standardised implementation of evidence-based nursing care practices. The findings provide evidence for the potential role of nursing-focused psychotherapeutic interventions in improving nursing-sensitive outcomes such as coping skills and self-esteem among individuals with GAD and may support efforts to expand access to such interventions.

The limited availability of psychotherapeutic interventions and restricted nurse authorisation in mental health services underscores the need for evidence-based approaches. To our knowledge, this is the first study evaluating the model for ineffective coping in outpatient psychiatric patients with GAD. However, prior to the wider integration of the model into routine clinical practice, its effectiveness should be confirmed through multicenter studies with larger and more diverse samples and longer follow-up periods.

Future studies should evaluate the long-term effects of the Psychotherapeutic Intervention Model in Nursing on ineffective coping among outpatients with GAD by incorporating follow-up assessments at 6 and 12 months. Comparisons between intervention and TAU groups, as well as between pharmacotherapy and different psychotherapeutic approaches, are needed. Where feasible, active control groups should be included to account for attention effects, and more rigorous blinding procedures should be implemented to reduce potential bias. Future research should also assess the cost-effectiveness of the intervention within the context of current psychiatric nurse workforce planning. In addition, the model should be re-evaluated using the most recent NANDA-I (2024–2026) and NOC (7th edition) classifications. Reporting intervention fidelity measures, such as session checklists and protocol adherence assessments, may further strengthen the reproducibility and methodological rigor of future studies. Evaluating the model across diverse care settings and patient populations will contribute to a stronger evidence base for its clinical application.

## Data Availability

The data that support the findings of this study are available on request from the corresponding author. The data are not publicly available due to privacy or ethical restrictions.

## References

[1] Chen TR, Huang HC, Hsu JH, Ouyang WC, Lin KC (2019). Pharmacological and psychological interventions for generalized anxiety disorder in adults: A network meta-analysis. Journal of Psychiatric Research.

[2] Boland R, Verduin ML, Ruiz P (2024). Kaplan & Sadock Psikiyatri.

[3] Boland R, Verduin ML, Ruiz P, Bozkurt A, Sarıkaya BB (2024). Kaplan & Sadock Psikiyatri. Kaygı Bozuklukları.

[4] Halter MJ (2022). Varcarolis Foundations of Psychiatric-Mental Health Nursing: A Clinical Approach.

[5] Wells A (1999). A metacognitive model and therapy for generalized anxiety disorder. Clinical Psychology & Psychotherapy.

[6] Kim Yong, Park Jong-Il, Park Tae-Won, Chung SangKeun, Yang Jong-Chul (2017). Stress Coping Strategies and Cognitive Characteristics of Somatic Symptom Perception in Patients with Generalized Anxiety Disorder. Anxiety and Mood.

[7] Badura-Brzoza K, Główczyński P, Dębski P, Błachut M, Skalski-Bednarz SB (2025). Ego-Resiliency and Coping Styles in Patients with Generalized Anxiety Disorder During the COVID-19 Pandemic. Medicina (Kaunas, Lithuania).

[8] Folkman S (2013). Stress: Appraisal and Coping. Encyclopedia of Behavioral Medicine.

[9] Huppert JD, Franklin ME, Barlow DH, Sadock BJ, Sadock VA, Ruiz P (2017). Anxiety disorders: cognitive-behavioral therapy. Comprehensive Textbook of Psychiatry.

[10] Slee A, Nazareth I, Bondaronek P, Liu Y, Cheng Z, Freemantle N (2019). Pharmacological treatments for generalised anxiety disorder: a systematic review and network meta-analysis. Lancet (London, England).

[11] Nutt D, Argyropoulos S, Hood S, Potokar J (2006). Generalized anxiety disorder: A comorbid disease. European Neuropsychopharmacology: the Journal of the European College of Neuropsychopharmacology.

[12] Ren L, Fan Y, Wu W, Qian Y, He M, Li X (2024). Anxiety disorders: Treatments, models, and circuitry mechanisms. European Journal of Pharmacology.

[13] Newman MG, Basterfield C, Erickson TM, Caulley E, Przeworski A, Llera SJ (2022). Psychotherapeutic treatments for generalized anxiety disorder: cognitive and behavioral therapies, enhancement strategies, and emerging efforts. Expert Review of Neurotherapeutics.

[14] Amaral RI, Weston FCL, Hirakata VN, Paz AA, Wesner AC (2022). Effectiveness and Efficacy of Therapeutic Interventions Performed by Nurses for Anxiety Disorders: A Systematic Review. Journal of the American Psychiatric Nurses Association.

[15] Cameron D, Kapur R, Campbell P (2005). Releasing the therapeutic potential of the psychiatric nurse: a human relations perspective of the nurse-patient relationship. Journal of Psychiatric and Mental Health Nursing.

[16] Hurley J, Lakeman R, Linsley P, Ramsay M, Mckenna-Lawson S (2022). Utilizing the mental health nursing workforce: A scoping review of mental health nursing clinical roles and identities. International Journal of Mental Health Nursing.

[17] Kappelin C, Carlsson AC, Wachtler C (2022). Specific content for collaborative care: a systematic review of collaborative care interventions for patients with multimorbidity involving depression and/or anxiety in primary care. Family Practice.

[18] Sampaio FMC, Sequeira C, Lluch Canut T (2017). Content Validity of a Psychotherapeutic Intervention Model in Nursing: A Modified e-Delphi Study. Archives of Psychiatric Nursing.

[19] Wheeler K (2014). Psychotherapy for the Advanced Practice Psychiatric Nurse: A How-To Guide for Evidence-Based Practice.

[20] Hartley S, Raphael J, Lovell K, Berry K (2020). Effective nurse-patient relationships in mental health care: A systematic review of interventions to improve the therapeutic alliance. International Journal of Nursing Studies.

[21] Herdman TH, Kamitsuru S, Lopes CT (2024). NANDA International Nursing Diagnoses: Definitions and Classification 2024–2026.

[22] Keil RMK (2004). Coping and stress: a conceptual analysis. Journal of Advanced Nursing.

[23] Wagner CM, Butcher HK, Clarke MF (2023). Nursing Interventions Classification (NIC).

[24] Moorhead S, Johnson M, Maas ML, Swanson E (2018). Nursing Outcomes Classification (NOC): Measurement of Health Outcomes.

[25] Shin JH, Choi GY, Lee J (2021). Identifying Frequently Used NANDA-I Nursing Diagnoses, NOC Outcomes, NIC Interventions, and NNN Linkages for Nursing Home Residents in Korea. International Journal of Environmental Research and Public Health.

[26] Johnson M, Moorhead S, Bulechek GM, Butcher HK, Maas ML, Swanson E (2011). NOC and NIC Linkages to NANDA-I and Clinical Conditions.

[27] Sampaio FMC, Araújo O, Sequeira C, Lluch Canut MT, Martins T (2018). A randomized controlled trial of a nursing psychotherapeutic intervention for anxiety in adult psychiatric outpatients. Journal of Advanced Nursing.

[28] Hopewell S, Chan AW, Collins GS, Hróbjartsson A, Moher D, Schulz KF (2025). CONSORT 2025 statement: updated guideline for reporting randomised trials. BMJ (Clinical Research Ed.).

[29] Aşkan Tuğba (2025). Evaluation of Psychotherapeutic Intervention Model in Nursing on Patients with Nursing Diagnosis of Ineffective Coping. [Doctoral Dissertation].

[30] Cuijpers P, Sijbrandij M, Koole S, Huibers M, Berking M, Andersson G (2014). Psychological treatment of generalized anxiety disorder: a meta-analysis. Clinical Psychology Review.

[31] Hurtado MM, Villena A, Vega A, Amor G, Gómez C, Morales-Asencio JM (2020). 'I have anxiety, but I have values and preferences' Experiences of users with generalized anxiety disorder: A qualitative study. International Journal of Mental Health Nursing.

[32] Bondarchuk O, Balakhtar V, Pinchuk N, Pustovalov I, Pavlenok K (2024). Coping with stressfull situations using coping strategies and their impact on mental health. Multidisciplinary Reviews.

[33] Ihara S, Katayama N, Nogami W, Amano M, Noda S, Kurata C (2024). Comparison of changes in stress coping strategies between cognitive behavioral therapy and pharmacotherapy. Frontiers in Psychiatry.

[34] Pozzi G, Frustaci A, Tedeschi D, Solaroli S, Grandinetti P, Di Nicola M (2015). Coping strategies in a sample of anxiety patients: factorial analysis and associations with psychopathology. Brain and Behavior.

[35] Mohamed S, Ewise H, Ali S (2023). Effect of Psychoeducational Training Program on Coping Skills, Spiritual Well-being, and Levels of Anxiety among Patients with Generalized Anxiety Disorder. Evidence-Based Nursing Research.

[36] Carl E, Witcraft SM, Kauffman BY, Gillespie EM, Becker ES, Cuijpers P (2020). Psychological and pharmacological treatments for generalized anxiety disorder (GAD): a meta-analysis of randomized controlled trials. Cognitive Behaviour Therapy.

[37] Craske MG, Stein MB (2016). Anxiety. Lancet (London, England).

[38] Fagan HA, Baldwin DS (2023). Pharmacological Treatment of Generalised Anxiety Disorder: Current Practice and Future Directions. Expert Review of Neurotherapeutics.

[39] O'Leary KB, Khan JS (2024). Pharmacotherapy for Anxiety Disorders. The Psychiatric Clinics of North America.

[40] Pulopulos MM, Hoorelbeke K, Vandenbroucke S, Van Durme K, Hooley JM, De Raedt R (2023). The interplay between self-esteem, expectancy, cognitive control, rumination, and the experience of stress: A network analysis. Current Psychology.

[41] Mathew B (2022). Effectiveness of psychological intervention package on anxiety and wellness level among patients with anxiety disorders. Journal of Family Medicine and Primary Care.

[42] Taylor TL, Montgomery P (2007). Can cognitive-behavioral therapy increase self-esteem among depressed adolescents? A systematic review. Children and Youth Services Review.

[43] Kunikata H, Yoshinaga N, Shiraishi Y, Okada Y (2016). Nurse-led cognitive-behavioral group therapy for recovery of self-esteem in patients with mental disorders: A pilot study. Japan Journal of Nursing Science: JJNS.

[44] Beattie S, Beattie D (2018). An investigation into the efficacy of a cognitive behavioural therapy group for low self-esteem in a primary care setting. The Cognitive Behaviour Therapist.

[45] Griffioen BT, van der Vegt AA, de Groot IW, de Jongh A (2017). The Effect of EMDR and CBT on Low Self-esteem in a General Psychiatric Population: A Randomized Controlled Trial. Frontiers in Psychology.

[46] Kolubinski DC, Frings D, Nikčević AV, Lawrence JA, Spada MM (2018). A systematic review and meta-analysis of CBT interventions based on the Fennell model of low self-esteem. Psychiatry Research.

[47] Yoshinaga N, Tanoue H, Hayashi Y (2022). Naturalistic outcome of nurse-led psychological therapy for mental disorders in routine outpatient care: A retrospective chart review. Archives of Psychiatric Nursing.

[48] Sledge WH, Moras K, Hartley D, Levine M (1990). Effect of time-limited psychotherapy on patient dropout rates. The American Journal of Psychiatry.

[49] Herdman TH, Kamitsuru S, Lopes CT (2021). NANDA International Nursing Diagnoses: Definitions and Classification 2021–2023.

[50] Moorhead S, Swanson E, Johnson M (2023). Nursing Outcomes Classification (NOC).

